# Associations of kidney injury markers with subclinical cardiovascular disease: the Multi-Ethnic Study of Atherosclerosis 

**DOI:** 10.5414/CN108668

**Published:** 2015-11-11

**Authors:** Meyeon Park, Michael G. Shlipak, Eric Vittinghoff, Ronit Katz, David Siscovick, Mark Sarnak, Joao A. Lima, Chi-yuan Hsu, Carmen A. Peralta

**Affiliations:** 1 University of California San Francisco, Department of Medicine, Division of Nephrology,; 2 San Francisco VA Medical Center, Division of General Internal Medicine,; 3 University of Washington,; 4 New York Academy of Medicine,; 5 Tufts Medical Center, and; 6 Johns Hopkins University School of Medicine, Baltimore, MD, USA

**Keywords:** kidney injury biomarkers, cardiovascular disease

## Abstract

No abstract available.

## Letter to the Editor 

Sir, – Novel urinary biomarkers have been found to indicate kidney tubular injury prior to a detectable rise in serum creatinine or decline in eGFR. These biomarkers may indicate chronic kidney tubular injury and progressive kidney disease [1, 2, 3]. Recent studies have shown that higher levels of kidney injury marker-1 (KIM-1) and albuminuria are associated with cardiovascular disease (CVD) and mortality in the general population [1] and in CKD patients [4], while higher levels of urine neutrophil gelatinase-associated lipocalin (NGAL) are also associated with cardiovascular events and mortality from heart failure [5]. The mechanisms underlying these associations are not well understood. 

Techniques to measure subclinical CVD may characterize the abnormal pathophysiology that precedes overt disease. Arterial stiffness is a candidate pathway that is associated with cardiovascular outcomes including death from coronary heart disease, myocardial infarction, and heart failure [6].Subclinical structural abnormalities including increased concentricity [7] are also associated with cardiovascular events and mortality [8]. 

We designed the current study to determine whether kidney injury detected by albuminuria and tubular biomarkers is associated with subclinical changes in vascular function and cardiac structure. We hypothesized that elevated levels of urinary albumin, NGAL, and KIM-1 would be associated with abnormalities in arterial stiffness and concentricity. 

We included participants from the Multi-Ethnic Study of Atherosclerosis (MESA), a cohort of individuals aged 45 – 85 free of CVD at baseline. Detailed methods are available in Supplemental Materials. Our cross-sectional analyses include MESA participants who were included in a case-control study [2] to evaluate the association of kidney injury biomarkers with incident kidney disease (n = 686). Briefly, cases were selected from persons without baseline CKD (defined as eGFR > 60 ml/min/1.73m^2^ by both creatinine and cystatin C) who either developed incident CKD (defined as reaching eGFRcys < 60 ml/min/1.73 m^2^ and having annual eGFRcys decrease of > 1 ml/min/1.73 m^2^ per year) and/or had rapid decline of kidney function (annual decline ≥ 1 ml/min/1.73m^2^). Controls were pair-matched on age, gender, race, baseline kidney function, and diabetes. Our primary exposures were levels of urine albumin to creatinine ratio (ACR), NGAL, and KIM-1 standardized to urine creatinine (cr). Our outcomes were subclinical CVD measures including functional measures of arterial elasticity, measured as large and small artery elasticity indices; and the structural measure of cardiac remodeling measured by concentricity, estimated as the ratio of left ventricular mass to left ventricular end-diastolic volume (LVEDV) [9]. 

Among the 686 MESA participants included in this study, mean age (SD) was 64.2 (9)****years, 48% were male, 31% had diabetes, 48% had hypertension. Median (IQR) NGAL/cr was 6.3 (1.9 – 22.4) ng/mg, KIM-1/cr 419.5 (242 – 741.4) pg/mg, and ACR 4.9 (2.9 – 10.5) mg/g. The prevalence of albuminuria (≥ 30 mg/g) was 11.6%. Mean baseline eGFR by the combined cys-cr equation was 78.8 (9.5) ml/min/1.73 m^2^. 

**Figure 1.**
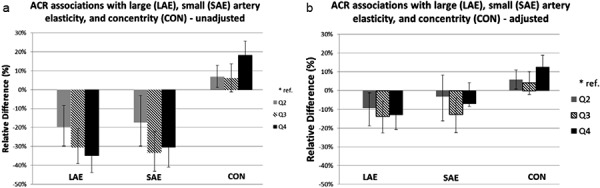
For LAE and SAE, negative relative difference indicates more severe disease. For CON, positive relative difference indicates more severe disease. Adjusted models include age, race, sex, systolic blood pressure, baseline eGFR. Models for each outcome also included the following covariates, according to predictor: Large artery elasticity. NGAL/cr: BMI, total and LDL cholesterol, diabetes, height, pulse. KIM/cr: BMI, total and LDL cholesterol, diabetes height, pulse. UACR: HDL cholesterol, diabetes, height, pulse. Small artery elasticity. NGAL/cr: height, pulse. KIM/cr: smoking, BMI, total and LDL cholesterol, diabetes, loop diuretics, height, pulse. UACR: BMI, total, LDL, and HDL cholesterol, triglycerides, diabetes, loop diuretics, height, pulse. Concentricity. NGAL/cr: smoking, BMI, total, LDL, and HDL cholesterol, triglycerides diabetes. KIM/cr: smoking, BMI, LDL cholesterol, diabetes, loop diuretics. UACR: smoking, BMI, total, LDL, and HDL cholesterol, triglycerides, diabetes.

Higher ACR was associated with more severe abnormalities of all three subclinical CVD outcomes in unadjusted models (Figure 1a). In unadjusted models, individuals in the highest quartile of ACR had 35% lower large artery elasticity when compared with the lowest quartile (relative difference (RD) –35%, 95% CI –43.8 to –24.8%). After adjustment for covariates including those that differed significantly between quartiles of albuminuria, associations were attenuated but significant (Figure 1b) (Table 1). For small artery elasticity, unadjusted models were statistically significant (Figure 1a), but adjustment attenuated these associations (Figure 1b) (Table 1). Those in the highest quartile of ACR had 18.2% higher concentricity (95% CI 11.3 – 25.5%) in unadjusted models (Figure 1a), which remained significant after adjustment (Figure 1b) (Table 1). 

Associations between NGAL/cr and all three CVD outcomes were not statistically significant in either unadjusted (data not shown) or adjusted models (Table 1). Similarly, associations between KIM-1/cr and all outcomes were not statistically significant (Table 1). Results were comparable when NGAL and KIM-1 were not standardized to urine creatinine (Supplementary Table 1). 

In a cohort of ethnically diverse individuals, we found that albuminuria was independently associated with worse arterial elasticity and cardiac remodeling. In contrast, associations of tubular markers with these outcomes were not significant after adjustment. Our finding that ACR is associated with subclinical CVD is consistent with prior reports, including in the MESA cohort [10].This is of particular interest because these associations were detected among persons with very low levels of ACR, and albuminuria is a known predictor of cardiovascular events. Our work expands on previous research by demonstrating associations of albuminuria with large arterial elasticity and concentricity independent of hypertension, systolic and diastolic blood pressure, and diabetes in a diverse population of individuals without significant preexisting CKD or CVD. 

Our observation that urine biomarkers of tubular injury are not associated with subclinical CVD is interesting because the physiology explaining associations between elevated urine tubular injury markers and long-term CV outcomes and mortality is not well understood. While our study did not find an association between tubular injury markers and outcomes, other studies have found that ACR is a stronger predictor of outcomes than tubular injury markers [1]. Mechanisms associated with albuminuria (such as endothelial dysfunction and inflammation) may be implicated in these associations, while those associated with tubular markers are not well understood [1]. The latter may include NSAID-induced nephropathy or other interstitial diseases, which may not be expected to contribute to cardiovascular disease pathogenesis. Our study is cross-sectional and cannot address mechanistic or causal relationships. Other limitations include single measurements of injury markers performed on stored urine samples and a relatively small sample size. 

In conclusion, we have found that kidney injury measured by ACR is associated with measures of subclinical CVD, while levels of tubular injury biomarkers NGAL and KIM-1 were not. Future studies of injury markers should focus on physiologic pathways to determine the mechanisms by which kidney injury markers are associated with CVD risk. 

## Funding 

NIH NIDDK K23 DK099238 (Park); K24 DK92291 (Hsu); RO1 AG027002 (MJS and MGS); 1K23DK082793 and by the Robert Wood Johnson Foundation (Harold J. Amos Award) (CAP). This research was supported by contracts N01-HC-95159, N01-HC-95160, N01-HC-95161, N01-HC-95162, N01-HC-95163, N01-HC-95164, N01-HC-95165, N01-HC-95166, N01-HC-95167, N01-HC-95168 and N01-HC-95169 from the National Heart, Lung, and Blood Institute and by grants UL1-TR-000040 and UL1-TR-001079 from NCRR. The authors thank the other investigators, the staff, and the participants of the MESA study for their valuable contributions. A full list of participating MESA investigators and institutions can be found at http://www.mesa-nhlbi.org. 

## Conflicts of interest 

None. 

## Supplementary material 

[Table SupplementalTable1]

## Detailed Methods

****Subjects****

We included participants from the Multi-Ethnic Study of Atherosclerosis (MESA) who also participated in the urinary biomarkers substudy [2]. Briefly, MESA is a large, ethnically diverse cohort of persons without cardiovascular disease (CVD), designed to study predictors of CVD. MESA recruited 6,814 men and women, ages 45 – 84 years, who self-identified as White, African-American, Hispanic, or Chinese-American. Details of recruitment and examinations have been described previously [11]. The baseline visit took place between July 2000 and September 2002. Additional details on the rationale and design for MESA are also available at http://www.mesa-nhlbi.org. 

Our cross-sectional analyses include the MESA participants who were included in a previously designed nested case-control study [2] to evaluate the association of kidney injury biomarkers with incident chronic kidney disease or rapid kidney function decline (n = 686). Cases were selected from persons without baseline CKD (defined as eGFR > 60 mL/min/1.73 m^2^ by both creatinine and cystatin C) who either developed incident CKD (defined as reaching eGFRcys < 60 mL/min/1.73 m^2^ and having annual eGFRcys decrease of > 1 mL/min/1.73 m^2^ per year) and/or had rapid decline of kidney function (annual decline ≥ 1 mL/min/1.73 m^2^). Controls were pair-matched on age, gender, race, baseline kidney function, and diabetes. 

****Exposures****

Urine albumin and creatinine were measured by MESA in a single morning urine sample by nephelometry and the rate Jaffe reaction, respectively, and expressed as albumin to creatinine ratio (ACR) in mg/g. Urinary KIM-1 and NGAL were measured in duplicate from previously frozen stored urine samples by a microbead-based assay from the baseline examination [12]. The inter- and intra-assay coefficient of variation for KIM-1 and NGAL was less than 8%. Urine creatinine concentrations were measured by the Jaffé assay using Randox Daytone Analyzer (Randox Laboratories Ltd., UK). The inter- and intra-assay coefficient of variation for creatinine was less than 3%. After collection, samples were refrigerated immediately and sent to lab the same day for processing and storage. Samples underwent two freeze-thaw cycles. All assays were performed in frozen serum specimens that were stored at –70 °C. 

****Outcomes****

Functional: arterial elasticity. To estimate the large (LAE) and small artery elasticity (SAE) indices, MESA used the HDI PulseWave CR-2000 Research CardioVascular Profiling Instrument (HDI, www.hdi-pulsewave.com) to acquire and analyze pulse waveforms from radial artery tonometry performed at the baseline examination. Briefly, the CR-2000 uses information the waveforms to make inferences about the elastic properties of the arterial tree, using diastolic pulse contour analysis and based on a third order, four element Windkessel modified model [13]. LAE and SAE measures have been shown to have high reproducibility in repeated measures [14, 15, 16, 17]. LAE and SAE estimates are independent predictors of incident hypertension [18], kidney function decline and CV events in MESA [18, 19]. 

Structural: The MESA MRI protocol has been described in detail previously [11, 20]. Our measurement for structural abnormalities was concentricity measured by cardiac magnetic resonance imaging (MRI), performed at the baseline examination. Concentricity has been shown to predict incident non-HF cardiovascular events more consistently than LV mass in the MESA cohort [9]. Cardiac concentricity was estimated as the ratio of LV mass to LVEDV [9] and is distinguished from concentric hypertrophy as determined by echocardiography as it does not use measurements of relative wall thickness. 

****Other variables****

Age, sex, race/ethnicity, level of education, and smoking (current, former, never) were ascertained by self-report using standardized questionnaires [11]. Height and weight were measured with participants wearing light clothing and no shoes with the use of calibrated scales. Body mass index (BMI) was calculated as weight in kilograms divided by height in meters squared. Blood pressure measurements were obtained using the Dinamap^®^ automated blood pressure device (Dinamap Monitor Pro 100^®^). Three sequential measures were obtained and the average of the second and third measurements was recorded. After a 12-hour fast, participants underwent phlebotomy to measure total cholesterol, high-density lipoprotein cholesterol, triglycerides, and glucose. Fasting blood was collected and stored at –70 °C until needed for the appropriate assays. High density lipoprotein (HDL) cholesterol was measured using the cholesterol oxidase cholesterol method (Roche Diagnostics, ‘Indianapolis, IN, USA). The Friedewald equation was used to calculate low density lipoprotein cholesterol [21]. Impaired glucose tolerance was defined by a fasting glucose level of 100 – 125 mg/dL without diabetes. Diabetes was defined as either a fasting glucose ≥ 126 mg/dl or use of oral hypoglycemic medication or insulin [22]. Use of antihypertensive medication (angiotensin converting enzyme inhibitors/angiotensin receptor blockers, diuretics) was recorded by study personnel. 

Serum creatinine was measured by rate reflectance spectrophotometry using thin film adaptation of the creatine amidinohydrolase method on the Vitros analyzer (Johnson & Johnson Clinical Diagnostics, Inc., Rochester, NY, USA) at the Collaborative Studies Clinical Laboratory at Fairview-University Medical Center (Minneapolis, MN) and calibrated to the Cleveland Clinic. Cystatin C was measured by means of a particle-enhanced immunonephelometric assay (N Latex Cystatin C, Dade Behring, Deerfied, IL, USA) with a nephelometer (BNII, Dade Behring) and calibrated for assay drift. We estimated the glomerular filtration rate (eGFR) with the use of a combined cystatin C and creatinine equation [23]. 

****Analysis****

We first described the baseline characteristics of the study participants by calculating means and standard deviations or medians and interquartile ranges for skewed variables. We used linear regression to evaluate univariate associations of injury biomarkers with covariates. We evaluated the cross-sectional associations between kidney injury markers and measures of subclinical cardiac vascular and structural predictors using multivariable regression models. Urine NGAL/cr, KIM-1/cr, and UACR were analyzed in quartiles to account for the non-linear distribution of the predictor variables. The outcomes of large and small artery elasticity and concentricity were log-transformed due to their skewed distributions. We used linear regression to compare each quartile of subclinical urine biomarker predictor to the reference quartile representing the lowest severity of injury. Beta coefficients were back-transformed to relative differences (RD), given as a percentage, for ease of interpretation. We used inverse probability weighting to account for case-control status. 

For all regression models, candidate covariates (chosen a priori) included age, race, sex, SBP, baseline eGFR (A-list), and smoking, BMI, total cholesterol, HDL, LDL, triglycerides, diabetes, ACEI/ARB, and diuretics (B-list). Candidate covariates were included in the final model using backward selection, with all A-list variables forced into the model and sequentially removing the B-list variables inducing the smallest change in the coefficient for the predictor, provided the change was less than 5%. Models for LAE and SAE also included heart rate and height forced into the models.

**Table 1.** Adjusted associations of UACR, NGAL/cr, and KIM-1/cr and outcomes of large artery elasticity, small artery elasticity, and concentricity.

	UACR			NGAL/cr			KIM-1/cr		
Large artery elasticity									
	Relative difference	95% CI	p-value	Relative difference	95% CI	p-value	Relative difference	95% CI	p-value
Q1	ref			ref			ref		
Q2	–9.5	–17.9, –0.3	0.0442	–4.4	–12.2, 4.1	0.2975	2.9	–6.9, 13.7	0.5727
Q3	–13.8	–21.7, –5	0.0028	1.6	–6.5, 10.3	0.715	1.9	–6.5, 10.9	0.6711
Q4	–13	–20.1, –5.3	0.0013	–1.5	–13.1, 11.7	0.8136	1.7	–9.4, 14.3	0.7707
Small artery elasticity									
	Relative difference	95% CI	p-value	Relative difference	95% CI	p-value	Relative difference	95% CI	p-value
Q1	ref			ref			ref		
Q2	–3.2	–14.6, 9.7	0.6071	–2.6	–13.5, 9.8	0.6677	–4.8	–15.2, 6.8	0.3995
Q3	–12.7	–21.3, –3.1	0.0107	–9.3	–21.1, 4.2	0.1678	–13.8	–25, –0.9	0.0362
Q4	–7.1	–18.4, 5.8	0.2691	–3.8	–15.4, 9.5	0.5589	3.3	–8, 16.1	0.58
Concentricity									
	Relative difference	95% CI	p-value	Relative difference	95% CI	p-value	Relative difference	95% CI	p-value
Q1	ref			ref			ref		
Q2	5.9	0.9, 11.1	0.0205	2.2	–3.3, 8	0.437	–0.2	–5.9, 5.8	0.947
Q3	4.2	–1.7, 10.4	0.1683	2.2	–3.8, 8.7	0.4744	–5.3	–10.8, 0.6	0.0795
Q4	12.6	6.4, 19.2	<0.0001	1.4	–3.5, 6.5	0.586	2.3	–3.5, 8.5	0.4416

All models adjusted for age, race, sex, systolic blood pressure, baseline eGFR. Models for each outcome also included the following covariates, according to predictor: Large artery elasticity. NGAL/cr: BMI, total and LDL cholesterol, diabetes, height, pulse. KIM/cr: BMI, total and LDL cholesterol, diabetes height, pulse. UACR: HDL cholesterol, diabetes, height, pulse. Small artery elasticity. NGAL/cr: height, pulse. KIM/cr: smoking, BMI, total and LDL cholesterol, diabetes, loop diuretics, height, pulse. UACR: BMI, total, HDL, and LDL cholesterol, triglycerides, diabetes, loop diuretics, height, pulse. Concentricity. NGAL/cr: smoking, BMI, total, LDL, and HDL cholesterol, triglycerides, diabetes. KIM/cr: smoking, BMI, LDL cholesterol, diabetes, loop diuretics. UACR: smoking, BMI, total, LDL, and HDL cholesterol, triglycerides, diabetes.

**Supplemental Table 1. SupplementalTable1:** Adjusted associations of NGAL, and KIM-1 and outcomes of large artery elasticity, small artery elasticity, and concentricity (not standardized to urine creatinine).

NGAL				KIM-1		
Large artery elasticity						
	Relative difference	95% CI	p-value	Relative difference	95% CI	p-value
Q1	ref			ref		
Q2	–3.8	–11.2, 4.1	0.3359	1.5	–5.6, 7.4	0.8374
Q3	1.9	–7.4, 12.1	0.7018	–9.2	–18.4, 1.1	0.0782
Q4	–2.3	–12.7, 9.3	0.6826	–2	–10.6, 7.4	0.6687
Small artery elasticity						
	Relative difference	95% CI	p-value	Relative difference	95% CI	p-value
Q1	ref			ref		
Q2	–0.9	–10.6, 9.8	0.8643	4.9	–6.9, 18.2	0.4299
Q3	–8.2	–20.3, 5.8	0.2363	–2	–12.8, 10.2	0.7373
Q4	–2.8	–13.7, 9.6	0.645	–1.3	–13.2, 12.2	0.8376
Concentricity						
	Relative difference	95% CI	p-value	Relative difference	95% CI	p-value
Q1	ref			ref		
Q2	4.4	–1, 10.1	0.1137	0	–4.4, 4.6	0.9897
Q3	2.8	–1.4, 7.2	0.1987	0.9	–4.2, 6.3	0.7252
Q4	1.9	–3.3, 7.3	0.4835	–1.3	–7.3, 5	0.6716

All models adjusted for age, race, sex, systolic blood pressure, baseline eGFR. Models for each outcome also included the following covariates, according to predictor: Large artery elasticity. NGAL: BMI, total and LDL cholesterol, diabetes, height, pulse. KIM-1: smoking, BMI, total cholesterol, diabetes, height, pulse. Small artery elasticity. NGAL: height, pulse. KIM-1: smoking, BMI, total and LDL cholesterol, diabetes, loop diuretics, height, pulse. Concentricity. NGAL: smoking, BMI, total, LDL, and HDL cholesterol, TG, diabetes, loop diuretics. KIM-1: smoking, BMI, LDL cholesterol, diabetes, loop diuretics.
